# Examining changes of adolescent physical habitus—a retrospective study of physical capital networks

**DOI:** 10.3389/fpsyg.2024.1306452

**Published:** 2024-02-08

**Authors:** Junyi Bian, Zubing Xiang, Xuechun Xiang

**Affiliations:** ^1^Department of Human Performance and Health Education, Western Michigan University, Kalamazoo, MI, United States; ^2^School of Physical Education, Guangzhou Sport University, Guangzhou, China; ^3^School of Physical Education, Chongqing University, Chongqing, China; ^4^Recreation and Sports Department of Trade Union of Chongqing University, Chongqing, China

**Keywords:** adolescent behavior, habitus, physical activity, physical habitus, social capital

## Abstract

**Introduction:**

In recent years, massive studies have examined sport from the standpoint of cultural capital. However, these studies have not examined cultural capital in relation to habitus within specific fields.

**Methods:**

This article utilized a multivariate latent growth curve model to analyze changes of participants’ physical habitus. Hypotheses concerning the form of growth in physical habitus, individual perceived capital influence differences in the common trajectory over time, and covariates influencing the growth of PH were tested.

**Results:**

Significant linear increases existed for perceived influence from family and school, while significant linear decreases existed for perceived influence from community. The second-order alternative format of the latent growth curve model indicated that associations among individual perceived influence differences could be adequately explained by a higher order physical habitus construct. Gender, personal health condition, family socio-economic status, and weekly time spent on physical activities significantly predicted initial levels of physical habitus, whereas family social economic status significantly predicted the rate of change in physical habitus simultaneously.

**Discussion:**

These findings not only support the participation in sport is constructed socially but also that individual efforts and contextual influences contribute to physical habitus. Furthermore, three types of perceived influences intertwined so much in simultaneity, instead of contributing to physical habitus across time.

## Introduction

Pierre Bourdieu’s theory of habitus, capital, and field has received substantial interest in the literature on sport participation (Hilgers and Mangez, 2014; Tiden et al., 2021; Dorsch et al., 2022). Bourdieu (1977) conceptualized sport as a social “field” that is a particular social space with its own unique characteristics, in which people have different attitudes and opinions toward sport. Given Bourdieu and Wacquant’s (1992) descriptions, “habitus” is not only a field that establishes certain socio-economic values and structures but also contains institutions instilling a worldview on people within it as to what is good and what is bad. The people then unconsciously internalize that worldview and then reproduce it within the habitus, thus strengthening the worldview within these institutions. This description of social “field” and “habitus” provides a framework for analysis of social behaviors as a fundamental protocol and helps us understand the reciprocal ways between thinking and actions, which always are integrated by individuals into an unconscious state Bourdieu conceptualizes as “habitus” (Threadgold, 2018).

When Bourdieu’s conceptualized framework is applied to contexts of physical activity participation (Gemar, 2020; Tiden et al., 2021), “physical habitus” (PH) is the extent of internalization of unconscious beliefs and values regarding physical activity. PH should be shaped through interactions among various social capitals, developed through repetitions of participation, and internalized to understand how to operate in a certain social context. The PH thus connects with multiple types of social capitals that are intertwined with each other—such as perceived influences from families, perceived influences from physical education in schools, and perceived influences from communities—which may directly or indirectly mold participants’ PH. In addition, the PH connecting the sport field and capital is expressed through the body in a way that responds to other agents in the field automatically and with predictability (Dukic et al., 2017; Smee et al., 2022). Given the above perspective, the PH also embodies the generative function of capital and a materialized form in terms of participants’ dispositions, which are desired indications to be looked for and quantified by physical education educators and health-promoting researchers (Strandbu et al., 2020; Zhang et al., 2022). However, this is difficult due to its dynamics and hiddenness. One potential solution to these discrepancies could predict participants’ PH indirectly based on the intertwined physical capital influences in relation to participatory behaviors.

Physical capital is a sub-species of social capital, which refers to the idea that one possesses connections with others in relation to physical activities, which can be embodied within specific social fields intercorrelated with other forms of capital (Koca et al., 2009). After Beijing Olympic Game in 2008, the software was emphasized from sport socialization perspective (Brownell, 2009). On the other hand, from the school perspective, as an example, Li et al. (2022) and Rick and Li (2023) reported that schools did not supply adequate resources for Chinese adolescent sport participation in developing their interests and skills. Obviously, various contextual influences are interrelated with participatory behaviors. However, most studies examining the interrelations between various contextual influences and sport participatory behaviors either measured several of contextual influences at a single time point (Truong et al., 2020; Volf et al., 2022) or explored a single contextual influence longitudinally (Farooq et al., 2020; Schmidlechner et al., 2021). It is possible that participatory behaviors are correlated with physical capital only at certain time points in childhood or adolescence. More importantly, it may be that different types of physical capital influences have different developmental trajectories over time. That means that the influence of some types of physical capital may increase over time, while others plateau or even decrease. For example, Dorsch et al. (2020) found that while there was an evident decline in parental involvement as children reach adolescence, this phenomenon in girls occurred earlier than boys.

Thus, the current study aims to (1) examine whether the levels of three perceived physical capital influences covary when they were measured repeatedly and determine whether the developmental trajectories of perceived physical capital influences covary over time and (2) examine the levels of PH through higher order LGCM and determine whether the range of change over time occurs. Specifically, in our tests of the personal characteristic’s effects on PH, three basic models were tested: an associative two-factor unspecified LGCM, a hierarchical factor-of-curves LGCM, and a hierarchical curve-of-factors LGCM before or after controlling for gender, self-evaluate healthy condition (SEHC), self-evaluate family social economic status (SEFSES), and weekly time spent on physical activities (WTSPA). Evidence of various perceived influence covariance, in either of these ways, would provide further justifications and a possible explanation for enabling some students, and restricting others, in physical activity participation (Chan et al., 2020; Smee et al., 2022). On this background, we will address the following questions:

1. How do the three types of physical capital influences covary over time as children progress into adolescence?
2. How does the PH change over time as children progress into adolescence?
3. Does the gender, self-evaluated health condition (SEHC), self-evaluated socio-economic status (SESES), and weekly time spent on physical activities (WTSPA) moderate the initial status and rate of change in PH over time?

## Literature review and hypothesis development

### Family influence on physical habitus

We know from Bourdieu’s theoretical framework about capital and habitus that the shaping of PH is a repeated socialization process, in which related physical capital is internalized into an active lifestyle. Children’s PH is typically started and cultivated within their families. Furthermore, a wide diversity of children’s health-enhancing behaviors, such as regular physical activity, often require parental engagement, encouragement, and influence (Dorsch et al., 2022). Although existing research stresses that there is a strong association between supportive family sport cultures and children or teenagers’ active involvement in sport (Knoester and Allison, 2022), there is still evidence of some decline in parental involvement as children reach adolescence (Dorsch et al., 2020). For instance, Dorsch et al. (2020) found that there was an evident decline in parental involvement as children reach adolescence. Furthermore, the decline in parental involvement in girls starts early, from 5th or 6th grade to 6th or 7th grade (Dixon et al., 2008). Those who assume that family influence should decrease as adolescents become more independent believe that peer influence from schools would then become more relevant to children’s physical activity and sport participation during adolescence (Gould et al., 2006; Dorsch et al., 2020). Yet, some adolescents’ self-reporting and quantitative statistical results suggest that parents continue to be more influential than peers in affecting children’s physical activity and sport participation during this stage of the life course (Li and Moosbrugger, 2021). Thus, not only is parental influence irreplaceable among various physical capitals in encouraging adolescent physical activity, but it is also important to consider the interplay between parental influence and other physical capitals. This is especially important in Chinese families, where due to Confucian culture’s influence and the longstanding linkage between family dignity and academic achievement, Chinese parents display a culture-specific parenting style, which places extremely high expectations on children’s academic and sport performance (Wang et al., 2022). This leads us to the following hypotheses:

*H1*: As children progress into adolescence, there is a trend toward an increase in perceived family influence in the child’s sport participation.

One other factor that needs to be considered is the initial family involvement in influencing sport participatory behaviors. Wheeler (2012) explained that parents believe themselves to be the catalysts behind their children’s initial involvement in sport. Much of the existing literature also finds parents responsible for initiating children’s sport participation (Johansen and Green, 2019; Tessitore et al., 2021). Specifically, the findings from a previous study showed that children enrolled in elementary school brought a diversity of participatory patterns from their homes that were molded further at school, which started to privilege certain PH over others (Ward and Griggs, 2018). Even though there was no required engagement in specialized sport in elementary school settings, there was still an emphasis on participation in basic physical activities such as running, throwing, and jumping. At this stage, the PH that was accumulated at home with their parents provided capital generative functions to those children who were actively involved in sport later. These children had thus already made progress toward cultivating an internalized PH which they embodied to display physiological, cognitive, social, and emotional traits their peers did not possess in the same combination.

*H2*: The initial status of perceived family influence in sport is positively associated with the rate of increase in internalized PH.

### School influence on physical habitus

There is a well-known assumption that physical education in school is not only often portrayed as a potentially significant vehicle for enhancing young students’ engagement with physically active recreation (Green, 2014; Tsangaridou, 2022) but that also significantly leads to greater engagement in sport later. Indeed, the profiles of the effects reported were different among studies in which various conceptual models were used (Choi et al., 2021), and the debate continues about the most suitable model to measure and interpret how physical education impacts student participatory behaviors later (Brickwood et al., 2019; Bruner and Martin, 2020). It advocates that the multidimensional concept of physical literacy be used in the education sector to embrace not only physical capacities and skills execution but also learning processes that instill confidence, motivation, and enjoyment in executing movement (WHO, 2018).

The existing Chinese physical education curriculum requirements from elementary, middle, to high school focus on guiding students to become skilled movers through repetition and rehearsal of technical skills. Furthermore, three stages of motor learning permeate the existing physical educational system. First, each child learns about correct and precise movement execution through an instructor’s demonstration, verbal instructions, and corrective feedback from an instructor. The students are frequently asked to perform decomposed parts of a movement, which they later rebuild in an isolated context. Second, the transfer stage emphasized more refinement of techniques in controlling the body. In this stage, the child begins to transfer their attention toward features outside of the body and toward the decomposed parts they made in the previous stage. Finally, the next stage guides students in purposefully achieving automaticity of movement, meaning that a well-refined movement can be done unconsciously. This pedagogical approach holds on the premise that the motor learning process is a gradual, linear process and reflects the continued elaboration of a mental model (Fitts and Posner, 1967). The advantage of this physical education pedagogical approach based on cognitive load theory is that it encourages instructors to ask their students to master techniques and their performance proficiently. In addition, in this approach, physical performance is easily judged and assessed by instructors depending on physical class criteria or rubrics. However, there are some disadvantages to this pedagogical approach in at least two aspects. First, instructors act as authoritative experts responsible for delivering objective motor skills and knowledge, resulting in a power imbalance between the instructors and students. This imbalance is more obvious in middle and high school. Second, the largest side effect of this approach is that it leaves little space for students to contribute to the learning process by searching and exploring their environments and adapting their actions by self-regulation, which are the core points toward building socialization in PH development.

An ecological dynamic approach means that as children discover information on how to move, they progressively refine their exploration so that they can detect richer and more reliable information which influences action. This transitory process is facilitated through individuals harnessing self-regulation tendencies that exist in all biological systems, influencing the passage from one organized state of the system to another (Kelso, 1995). This leads us to the following hypothesis:

*H3*: A increasing trend of perceived school influence on sport participation exists as children progress into adolescence, while the rate of increase held by the students who have a higher initial perceived school influence will be slower.

### Community influence on physical habitus

Building environment is defined as social infrastructure and services in local communities that would play important roles in influencing children and adults’ social engagement (Orpana et al., 2016). Previous research has stressed that building environments offer different spaces and opportunities to fortify participatory behaviors in sport (Hanlon et al., 2022). However, some researchers argue that a gradual shift in how individuals interact with one another has occurred, resulting in decreased levels of community influence and less meaningful social interactions within communities (Shams-White et al., 2021). Community influence is defined by feelings of belonging and collective identity, which are derived from ongoing and meaningful contact with others (Slomski, 2019). From this perspective, participating in sport within a positive community setting could create a feeling of influence and belonging that those members of a community share. Through meaningful community influence, participation in sport within the neighborhood may act as a catalyst for the building of participants’ PH.

Currently, China’s economic development pattern is transforming from high-speed growth to high-quality development, which means the improvement of the living environment and the satisfaction of residents’ expectations for a better life. In this aspect, sport development needs to find a solution for the existing contradiction between people’s health needs and the lack of sport facilities. Specifically, after implementing the Healthy China 2030 Plan in October 2016, community parks, public green avenues, and grace spaces have provided a good starting point for providing the required hardware to build community influence. We know from social capital theory that community influence resources embedded in social ties must first be accessed and then mobilized to provide benefits (Lin et al., 2019; Bian and Xiang, 2023). Mobilization is thus key to activating information, knowledge, community influence, or other resources that have been conceptualized as social capital. Thus, in addition to the required hardware for building community influence, the “software”—or extra psychological factors such as feelings of belonging and collective identity—is also important for children and adolescents to build their PH, and this still needs upgrading in China. This leads us to the following hypothesis:

*H4*: A decreasing trend of perceived community influence on sport participation exists as children progress into adolescence, while the rate of decrease held by the students who have a higher initial status will be slower.

Finally, we can develop a hypothesis about the overall development of PH among children:

*H5*: An increasing trend of PH exists as children progress into adolescence, while the rate of increase held by students who have a higher initial status will be slower.

### Conceptual framework

**Figure 1:**
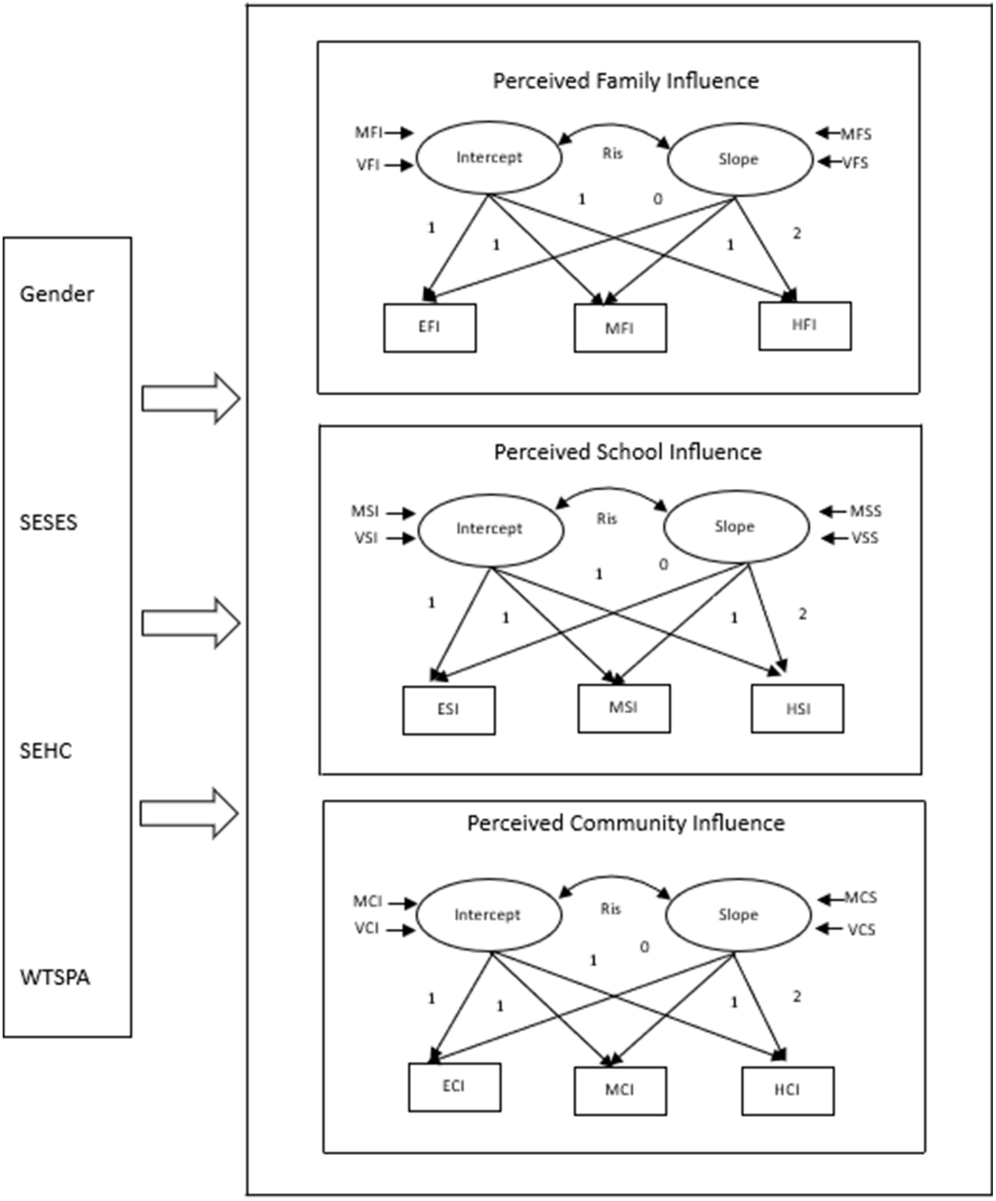
Representation of the conceptual framework. EFI, elementary school family sport influence; MFI, middle school family sport influence; HFI, high school family sport influence; ESI, elementary school sport influence; MSI, middle school sport influence; HSI, high school sport influence; ECI, elementary school community sport influence; MCI, middle school community sport influence; HCI, high school community sport influence; MFI, mean of family sport influence intercept; MFS, mean of family sport influence slope; VFI, variance of family sport influence intercept; VFS, variance of family sport influence slope; MSI, mean of school sport influence intercept; MSS, mean of school sport influence slope; VSI, variance of school sport influence intercept; VSS, variance of school sport influence slope; MCI, mean of community sport influence intercept; MCS, mean of community sport influence slope; VCI, variance of community sport influence intercept; VCS, variance of community sport influence slope; SESES, self-evaluated family social economic status; SEHC, self-evaluated health condition; WTSPA, weekly time spent on physical activities; Ris, correlation coefficient between intercept and slope.

Figure 1 presents our theoretical framework. First, this framework envisioned that it may be that the different types of perceived influences on PH have different developmental trajectories over time (i.e., family, school, and community). Second, in addition to the individual perceived influences on PH, this framework also highlighted that the perceived influence on PH from family, school, and community was correlated within a specific sport culture at certain time points in childhood or adolescence. That means that the influence of some types of sport influence may increase over time, while others plateau or even decrease. Given this background, we will address the following core question: How does youths’ perceived sport influence from their family, school, and community on changes of PH over time as children progress into adolescence?

## Method

### Research design and participants

Based on our research goals, our study employed the retrospective non-experimental research design, investigating Chinese collegiate students’ perceived influences regarding sport from family, school, and community that impact PH mutually and indirectly in 2022. The participants were sampled using a purposive selection method by school type and composed of 1,500 college students from 16 universities, representing all types of colleges and universities in Sichuan province in China. Chongqing University Institute Review Board approved this study. When the questionnaire was created by Wen Juan Xing (WJX) which is a popular survey tool online in China, the informed consent form was posted on the introduction page. The aim was to give the participants an overview of the purpose of this study, to inform them of the procedures protecting research participants’ identity, and data disposal procedures after the completion of the online survey. Considering fraudulent response online, the following strategies were deployed to secure participants’ responses. The first step is to utilize screening questions which can provide researchers with some assurance that participants in the sample are representative of the population of interest and that they possess sufficient knowledge or experience related to the task being examined. Second, participants are online and potentially browsing the web, performing multiple tasks, interacting with their phone, or doing a myriad of other activities simultaneously while completing a survey. Thus, two attention check questions, which automatically ended the survey if a wrong response was provided, were also included in the survey. Finally, the questionnaire was created by WJX and the survey link was dispatched by their faculties in the physical education departments. Finally, the survey was completed electronically.

To be sure of the desired power of 0.80 for related coefficients, an unspecified latent growth curve model (LGCM) over three time points without covariates was treated as a multivariate model with correlated relationships among perceived influences. Furthermore, depending on previous literature findings (Thomas et al., 2019) about the relationship between perceived influence from family and school was more correlated than the relationship between perceived influence from school and community, and the presumed correlation between perceived influence from school and family, the presumed correlation between the school and community, and the presumed correlation between family and community were set 0.50, 0.30, and 0.15, respectively. It was noteworthy that all latent intercept mean and slope mean were set 0.50 and 0.10. At last, a varied sample size from 100 to 1,000 was simulated by Monte Carlo in R package SIMSEM and LAVVAN (Moshagen, 2021; Orcan, 2021), and a minimum sample size of 887 is required to obtain desired power of 0.08 at *α* = 0.05 significant level. Considering no response conditions, 1,500 questionnaires were distributed and ultimately a valid sample of 934 individuals occurred.

### Procedures

Participants completed a series of self-report questionnaires assessing their PH indirectly through perceived physical capital influence around them. The physical capital influences consist of perceived influences from family, school, and community, respectively. The demographic characteristics were surveyed as well. To increase the likelihood of valid reporting by participants, a certificate of confidentiality was obtained from the Guangzhou Sport University that precluded the privacy disclosure of participants’ data. Informed consent procedures also indicated that no one except the authors would be informed of participants’ responses.

### Measurements

#### PH

Physical habitus was measured from perceived influence regarding sport from family, school, and community. Measures of perceived influence from family (FI) were constructed from three items assessing the frequency of family member’s influence. The three-item survey question asked whether “I always participate in sports with my family members,” “My family often teaches me about sports,” and “My family is often involved in sports.” Measures of perceived influence from school (SI) were constructed from three items assessing satisfaction toward what the school offered. The three-item survey question asked whether “Sports utilities that my elementary school provides meet my desire for physical activities,” “My school often provides organized sports for us after school,” and “My school is very active in sports.” Measures of perceived influence from the community (CI) were constructed from three items assessing sport utility convenience the communities offered. The three-item survey question asked whether “There are sufficient sports facilities near the place of residence,” “My neighborhood is very active in sports,” and “My neighborhood always accepts new playmates.” A five-point scale was utilized to measure the extent to which the respondents responded. The five levels of the scale are 1 (Strongly Disagree), 2 (Somewhat Disagree), 3 (Neither agree nor disagree), 4 (Somewhat Agree), and 5 (Strongly Agree). All items were summed to create an overall influence.

#### Self-evaluated social economic status (SESES)

SESES was measured from household annual income assessing family economic status by a 5-point scale from bad to excellent.

#### Self-evaluated health condition (SEHC)

SEHC was measured from an item assessing own health condition on a 5-point scale from bad to excellent.

#### Weekly time spent on physical activities (WTSPA)

WTSPA was measured from two items: one assessing how much time respondents spend on physical activities during workdays and the other assessing how much time respondents spend on physical activities on weekends. The two items were summed to create an overall WTSPA.

### Instrument validity and reliability

#### Validity

The following steps were employed to secure the instrument’s validity. (1) The measures of perceived influence regarding sport were adapted from a previous research study (Kim and James, 2019), which allowed minimal modifications to fit into the measured contents. (2) An expert committee related to sport participatory behaviors was established to assess the content validity of the survey instruments, processing four rounds of expert checks. (3) A pilot survey was conducted to ensure the tone of the wording, and the format and flow of the survey were easy for participants to follow and respond to. (4) To investigate the factorial structure of the specific perceived influence items and validate the construct of perceived influence, a one-factor confirmatory factor analysis (CFA) was conducted in R 4.40 with the 934-response dataset using a robust maximum likelihood estimation corresponding to results shown in Table 1.

**Table 1:** One-factor CFA results for each item of FI, SI, and CI (*n* = 934).

Stage	Construct	Items	KMO	β	CR	AVE (%)
Elementary	Family influence	EFI-1	0.742	0.757**	0.877	83.79
		EFI-2		0.862**		
		EFI-3		0.894**		
Middle	Family influence	MFI-1	0.708	0.715**	0.850	80.70
		MFI-2		0.847**		
		MFI-3		0.859**		
High	Family influence	HFI-1	0.705	0.773**	0.883	84.41
		HFI-2		0.853**		
		HFI-3		0.907**		
Elementary	School influence	ESI-1	0.772	0.757**	0.877	83.79
		ESI-2		0.862**		
		ESI-3		0.894**		
Middle	School influence	MSI-1	0.788	0.715**	0.850	80.70
		MSI-2		0.847**		
		MSI-3		0.859**		
High	School influence	HSI-1	0.735	0.773**	0.883	84.41
		HIS-2		0.853**		
		HIS-3		0.907**		
Elementary	Community influence	ECI-1	0.752	0.790**	0.896	86.01
		ECI-2		0.906**		
		ECI-3		0.885**		
Middle	Community influence	MCI-1	0.750	0.854**	0.927	89.88
		MCI-2		0.919**		
		MCI-3		0.923**		
High	Community influence	HCI-1	0.750	0.849**	0.922	89.29
		HCI-2		0.916**		
		HCI-3		0.914**		

β, standardized factor loading; CR, composite reliability coefficient; AVE, average variance extracted. **p < 0.01.

#### Reliability

Two types of reliability tests including test–retest reliability and internal consistency reliability were conducted. First, to check the test–retest reliability of specific perceived influence regarding sport, the instrument was administered twice to the same participants (*n* = 60) within a 1-week interval. The test–retest reliability coefficient was calculated via the correlation coefficients between the two sets shown in Table 2. Second, the internal consistency reliability of perceived influence items was examined via assessing the interrelationships among items with the 934-response data. Internal consistency reliability coefficients Cronbach’s alpha and McDonald’s omega were examined in SPSS. 28. Hayes and Coutts (2020) suggested that the correlation coefficient values, Cronbach’s alpha and McDonald’s omega, at 0.7 or above would indicate acceptable internal consistency reliability in a research setting.

**Table 2:** Reliability results for FI, SI, and CI.

		Test–Retest (*n* = 60)	Cronbach’s alpha (*n* = 934)	McDonald’s omega (*n* = 934)
FI	Elementary	0.826**	0.906	0.907
	Middle	0.834**	0.918	0.916
	High	0.801**	0.928	0.928
SI	Elementary	0.799**	0.888	0.892
	Middle	0.734**	0.904	0.910
	High	0.813**	0.911	0.914
CI	Elementary	0.823**	0.911	0.914
	Middle	0.734**	0.943	0.945
	High	0.854**	0.950	0.951

** stands for correlation coefficient is significant at *α* = 0.05 level, FI stands for family influence, SI stands for school influence, and CI stands for community influence.

### Statistical procedures

Data screening was completed before descriptive analyses (i.e., means and standard deviations, percentages, and frequency) were conducted using SPSS. Outliers were also removed. Then, descriptive analysis was performed, followed by tests of multivariate normality (chi-square probability plot) to ensure the validity of the model. A series of multivariate latent growth curve models (LGCM) were the primary statistical technique for the present study. The following specifies the modeling process.

Initially, an associative two-factor unspecified LGCM (M1) was estimated to determine the form of growth and the pattern of associations that existed among the growth parameters of three types of perceived influences. For instance, the first common factor is labeled the intercept and represents individual perceived family influence differences occurring in the elementary school stage at the group level over time. Furthermore, the intercept is a constant for any individuals across time and represents the average value of intercepts represented by MFI. Similarly, the variance of intercept, represented by VFI, stands for a collection of individual intercepts that characterize an individual’s growth curve.

Second, to test the degree to which the relationships among the growth factors can be described by a higher order construct, the M1 was re-estimated as a hierarchical factor-of-curves LGCM (M2). The higher order model follows a structure such as the first-order associative two-factor unspecified LGCM. However, the covariances among the first-order factors are hypothesized to be explained by the higher order factors: intercept and slope of PH, respectively.

Third, considering the simultaneity and covariance of observed items measuring perceived respective influence occurred in elementary, middle, and high school, an alternative hierarchical curve-of-factors LGCM (M3) was employed. In fitting the M3, unique covariances for each observed item over time are allowed to covary and are included mainly to improve the goodness of fit of the model. The M3 explicitly requires a condition of factor pattern invariance where common factor pattern elements are required to be equal over time. The covariances among the first-order common factors are hypothesized to be explained by the higher order growth curve parameters: intercept and slope of PH.

Furthermore, a model considered a satisfactory fit should meet a set of criteria. For the absolute index, chi-square statistics and the number of freely estimated parameters (the model degree of freedom) are often considered; a standardized root mean square residual (SRMR) value of 0.000 indicates perfect model-data fit. Regarding the parsimony correction index, the root mean square error of approximation (RMSEA) estimate is viewed as an “error of approximation” index (Brown, 2015, p. 71). The RMSEA estimate and the corresponding 90% confidence limit of less than 0.10 indicate excellent model-data fit. Akaike’s information criterion (AIC) incorporates the number of parameters in the model, which is often employed for non-nested model selection. The better model is determined as the one with the smallest AIC value. Similar to AIC, the Bayesian information criterion (BIC), known as the Schwarz Bayesian Criterion, is also used for non-nested model selection. The smaller the BIC value, the better the model-data fit (Brown, 2015). With respect to comparative fit indices, the comparative fit index (CFI) has a range of possible values between 0.000 and 1.000. The closer to 1.000, the better the model-data fit; the Tucker–Lewis Index (TLI) may fall outside of the range of 0.000 to 1.000, but the closer to 1.000, the better the model-data fit.

## Results

### Demographic statistics for the research sample

A total of 1,500 respondents participated in the current study. After examining each individual response and screening the raw data, a total of 566 were removed due to its incompleteness and dissatisfaction for the screening questions and attention check. Finally, the researchers retained 934 usable surveys for the data analysis. Of the 934 respondents, there were 55.00% female respondents (*n* = 514) and 45.00% male respondents (*n* = 420). Approximately 22.30% (*n* = 209) of the participants’ mothers had a higher education attachment, while 24.30% (*n* = 227) of the participants’ fathers had a higher education attachment. Regarding the job category, over half of the participants’ mothers (*n* = 621, 66.4%) reported that they were full-time employee, while the corresponding value for participants’ fathers was 62.8% (*n* = 587). Furthermore, the descriptive statistics for the observed variables utilized on LGCM are shown in Table 3.

**Table 3:** Descriptive statistics for the observed variables utilized on LGCM.

	Family influence			School influence			Community influence		
	E	M	H	E	M	H	E	M	H
V1	1.000								
V2	0.796	1.000							
V3	0.766	0.871	1.000						
V4	0.604	0.615	0.554	1.000					
V5	0.585	0.764	0.679	0.753	1.000				
V6	0.486	0.647	0.658	0.662	0.804	1.000			
V7	0.578	0.667	0.627	0.509	0.617	0.546	1.000		
V8	0.606	0.758	0.723	0.512	0.698	0.644	0.790	1.000	
V9	0.565	0.710	0.734	0.501	0.664	0.667	0.737	0.856	1.000
	Female	Male	*M*	*SD*	Skewness	Kurtosis			
Gender	514	420							
SESES			−0.011	0.959	−0.640	2.415			
SEHC			2.712	1.841	0.246	−1.829			
WTSPA			214.228	196.614	1.887	1.796			

Correlation matrix is in the triangle; means, standard deviation, kurtosis, and skewness are presented in the bottom rows of the matrix; E, elementary school; M, middle school; H, high school; SESES, self-evaluated family social economic status; SEHC, self-evaluated health condition; WTSPA, weekly time spent on physical activities.

### Associative two-factor unspecified LGCM

Initially, an associative two-factor unspecified LGCM (M1) was estimated to determine the form of growth and the pattern of associations that existed among the growth parameters of the three types of perceived influences. The model depicted in Figure 1 represents an associative two-factor unspecified LGCM where the basic parameters describe a systematic pattern of individual differences in change over time. An associative two-factor unspecified LGCM allows for the assessment of relationships among the individual different parameters for perceived influence from family, school, and community and allows for the estimation of means, variances, and covariances for the growth of each influence. Model fitting procedures for the associative two-factor unspecified LGCM [χ^2^ (23, *N* = 834) = 363.749, *p* < 0.001, CFI = 0.954, TLI = 0.928, SMRE = 0.072, RMSEA = 0.086, AIC = 26294.763, BIC = 26441.276, sample-size-adjusted Bayesian (ABIC) = 26342.831] suggested that an associative unspecified LGCM representation of the various perceived influence was tenable.

The estimated parameters shown in Table 4 indicated a significant average mean in each perceived influence and significant growth at the group level. Estimated effects of initial perceived influence occurred in elementary school on growth pattern occurred in the high school stage, and all these coefficients are significant. Furthermore, the variances of intercepts and slopes for perceived influence from family, school, and community are all significant, indicating that significant individual variation existed in the development of these influences.

**Table 4:** Summary of correlations among three types of influence and parameters for the associative unspecified LGCM.

	Perceived family influence		Perceived school influence		Perceived community influence	
	Intercept	Slope	Intercept	Slope	Intercept	Slope
Family influence I	1.000					
Family influence S	0.008**	1.000				
School influence I	0.749**	0.025**	1.000			
School influence S	0.048**	0.137**	−0.225**	1.000		
Community influence I	0.795**	0.283**	0.650**	0.065**	1.000	
Community influence S	0.178**	0.459**	−0.007**	0.705**	−0.009**	1.000
Mean	6.863	0.023	6.446	0.105	7.078	−0.074
*t* value	97.241	3.561	91.064	1.962	97.963	−2.574
Variance	0.852	0.025	0.786	0.074	0.806	0.047
*t* value	17.393	4.197	17.207	9.034	17.275	6.799

**stand for correlation is significant at *p* < 0.05, *t* value > 1.96 is significant at *p* < 0.05 (two-tailed), I means intercept, and S means slope.

Table 4 also presents the correlations among the intercepts and slopes for the three types of perceived influence in this model. The intercepts and slopes of perceived influence from family, school, and community were all significantly correlated. Perceived school influence and community influence shared a common developmental trend which was different from perceived influence from family. The correlations supported hypothesized associations among the individual difference parameters for the various perceived influences (*H1*, *H2*, *H3*, and *H4* in *RQ1*).

### Hierarchical factor-of-curves LGCM

To test the degree to which the relationships among the growth factors can be described by a higher order PH construct, the associative two-factor unspecified LGCM was re-estimated as a hierarchical factor-of-curves LGCM (M2). The higher order model follows a structure such as the first-order multivariate LGCM. However, the covariances among the first-order factors are hypothesized to be explained by the higher order factors: intercept and slope of PH. Model fitting indexes for the hierarchical factor-of-curves LGCM [χ^2^ (34, *N* = 834) = 472.349, *p* < 0.001, CFI = 0.941, TLI = 0.937, RMSEA = 0.089, AIC = 26381.363, BIC = 26475.887, ABIC = 26412.374] suggested that a higher order common factor representation of the various influences was tenable. Parameter estimates for the hierarchical factor-of-curves model indicated significant mean levels in the common intercepts (MPHI = 5.619, *t* = 61.887, and the trajectory, MPHS = 0.287, *t* = 17.776). Individual differences in the higher order factors were significant with estimated variances of VPHI = 3.154, *t* = 14.891, and VPHS = 0.217, *t* = 10.886. Furthermore, all common hierarchical factor-of-curve coefficients were significant. In addition, the relationship between PH intercept and slope (Ris = 0.110, *t* = 2.840) indicates that the higher the initial PH level, the faster the increase in PH. In addition, the higher order factor intercept accounted for approximately 79.20, 70.60, and 75.00% of the variation in the first-order intercepts for perceived influence from family, school, and community, respectively. Similarly, the higher order factor slope accounted for approximately 73.45, 64.60, and 99.70% of the variation in the first-order trajectories changes for perceived influence from family, school, and community, respectively.

To test the degree to which there is consistency in the influence of the individual characteristics on the perceived influence, the factor-of-curves LGCM was re-identified to include the effects of the individual characteristics (gender, self-evaluated health condition, self-evaluated family social economic status, and weekly time spent on physical activities) on the second-order common factors. Model fitting procedures for the factor-of-curves LGCM resulted in an adequate fit of the model to the data [χ^2^ (62, *N* = 834) = 566.293, *p* < 0.001, CFI = 0.933, TLI = 0.922, RMSEA = 0.099, 90% CI [0.091, 0.106], SRM = 0.044, AIC = 26310.893, BIC = 26443.228, ABIC = 26354.309], suggesting that a higher order common factor representation of the various influence was tenable.

### Hierarchical curve-of-factors LGCM

**Figure 2:**
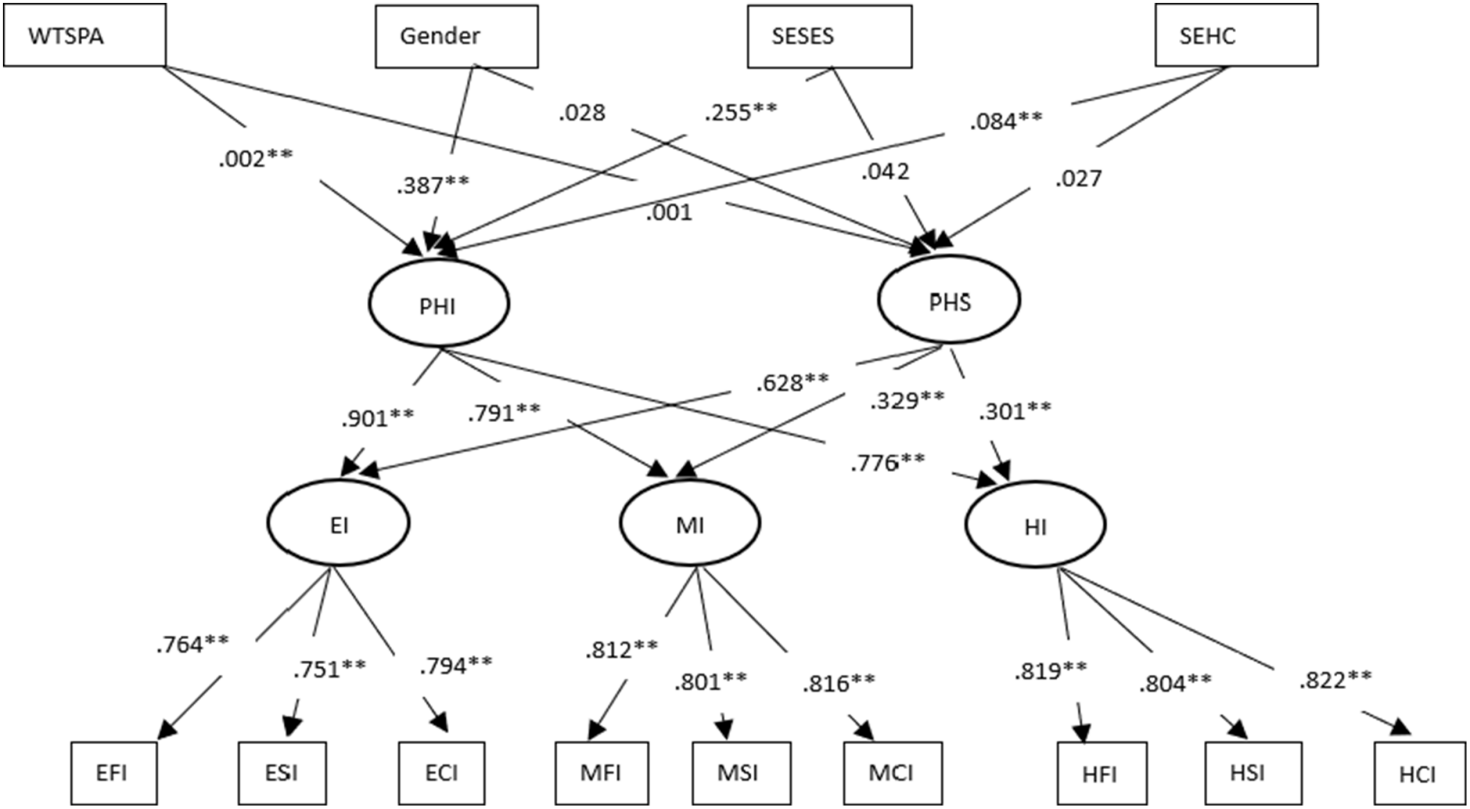
Summary of coefficients in hierarchical factor-of-curves LGCM (M3). EFI, elementary school family sport influence; ESI, elementary school sport influence; ECI, elementary school community sport influence; MFI, middle school family sport influence; MSI, middle school sport influence; MCI, middle school community sport influence; HFI, high school family sport influence; HSI, high school sport influence; HCI, high school community sport influence; EI, elementary school sport influence; MI, middle school sport influence; HI, high school sport influence; PHI, physical habitus intercept; PHS, physical habitus slope; WTSPA, weekly time spent on physical activities; SESES, self-evaluated family social economic status; SEHC, self-evaluated health condition.

Although the hierarchical factor-of-curves LGCM appeared to provide an adequate fit of the model to the data, we also tested the alternative hierarchical curve-of-factors LGCM (M3). The model represents the hierarchical curve-of-factors LGCM. Fitting the curve-of-factors LGCM resulted in the following indexes of fit [χ^2^ (30, *N* = 834) = 200.662, *p* < 0.001, CFI = 0.977, TLI = 0.969, RMSEA = 0.088, 90% [0.077, 0.099] SRMR = 0.048, AIC = 26123.675, BIC = 26251.284, ABIC = 26165.541], suggesting that a higher order common factor representation of the various influence was more fitting the data comparing to M1 and M2 due to lower SRME, AIC, and BIC values. Parameter estimates for the curve-of-factors model indicated significant mean levels in the intercept (MPHI = 4.075, *t* = 94.034) and trajectory changes (MPHS = 1.419, *t* = 66.535) of the common factors (*H_5_ in RQ2*). Individual differences in each higher order growth factor were significant with estimated variances of VPHI = 3.023, *t* = 15.889, and VPHS = 0.393, *t* = 7.778. Common factor loadings were all significant and are presented in Figure 2. In addition, the higher order factor intercept in the curve model accounted for approximately 72.00, 99.80, and 92.10% of the variation in the first-order perceived influence from elementary, middle, and high school, respectively. Similarly, 60.03, 61.73, and 65.97% of the variation in observed variables testing perceived influences in different stages, respectively, were accounted for by higher order factor slope in the growth curve model.

Model fitting procedures for the curve-of-factors LGCM when the effects of the individual characteristics were specified resulted in the following indexes of fit [*χ*^2^ (59, *N* = 934) = 349.516, *p* < 0.001, CFI = 0.961, TLI = 0.953, RMSEA = 0.077 with a 90% *CI* [0.069, 0.085], SRMR = 0.045, AIC = 26100.117, BIC = 26246.630, ABIC = 26148.184], suggesting that a hierarchical curve-of-factors representation of the various influence was tenable. Combined, covariates accounted for approximately 15.00 and 8.90% of the variation in the common intercept and slope, respectively.

Table 5 presents the standardized effects of the individual characteristics on the common factors representing the intercept and slope of the first-order repeated measures of perceived influence. This analysis resulted in exactly the same significant personal predictors the hierarchical factor-of-curves model demonstrated (Table 5). Gender, family social economic status, self-evaluated health condition, and weekly time spent on physical activities significantly impacted the PH intercept measured by perceived influence repeatedly. Family socio-economic status significantly impacted the common developmental trajectory growth of PH (RQ3).

**Table 5:** Personal characteristic effects on mean and growth of PH in factor-of-curves LGCM.

	Factor-of-curves LGCM (M2)			
Covariates	PH intercept		PH slope	
	*β*	*t*	*β*	*t*
Gender	0.391	3.007	0.026	0.609
SESES	0.290	4.272	0.042	2.040
SEHC	0.091	2.546	0.021	1.764
WTSPA	0.002	6.016	0.001	0.711

	Curves-of-factors LGCM (M3)			
	*β*	*t*	*β*	*t*
Gender	0.387	3.137	0.028	0.668
SESES	0.255	3.968	0.042	2.241
SEHC	0.084	6.208	0.027	1.414
WTSPA	0.002	6.208	0.001	0.005

*t* values > 1.96 are significant (two-tailed) at *p* < 0.05; SEHC, self-evaluate healthy condition; SEFSES, self-evaluate family social economic status; and WTSPA, weekly time spent on physical activities.

## Discussion

The main purpose of this study was to examine whether the levels of three perceived physical capital influences covary when they were measured repeatedly, determine whether the developmental trajectories of perceived physical capital influences covary over time, further explore the levels of PH through higher order LGCM, and determine whether the developmental trajectories of PH change over time, before or after controlling for gender, self-evaluate healthy condition (SEHC), self-evaluate family social economic status (SESES), and weekly time spent on physical activities (WTSPA).

Based on the analysis of the actual participants’ responses, a proposed research model was presented, and it was found that significant developmental increases existed in perceived influences from family and school, answering the H_1_, H_2,_ and H_3_, and significant decreases existed in perceived influences from community, answering the H_4_. The model also indicated that there was an upward trend in PH as children progressed into adolescence, answering the H_5_.

First, the findings from the associative two-factor unspecified LGCM (M1) are consistent with other literature (Forsell and Polman, 2020; Mastromartino et al., 2022) indicating that interconnections existed among perceived influences from family, school, and community but go beyond existing analyses of correlations at a single time point by examining correlations among various initial levels of perceived influences as well as their rates of change over time. The results show that initial levels of perceived family influence, perceived school influence, and perceived community influence are intercorrelated positively. Moreover, individual differences in the developmental trajectories for those three influences were positively and significantly related. These correlations justify a single increase in perceived influence contributing to both mean level and rates of change in PH. This is a strong indication of what earlier studies have described as a normative pressure from parents and schools to take part in sport (Johansen and Green, 2019). As an example, the vast majority of 14- to 15-year-olds in Strandbu et al.’s (2017) focus-group study, including those who no longer took part in organized sports, believed that their parents wanted them to participate. Furthermore, the current study also indicated that youths’ perception of the influence that sports play in their middle and high schools decreases during the teenage years, which is consistent with Strandbu et al.’s (2020) report. The fact that many Chinese parents and teachers pay more attention to keeping their young teenagers and students involved in academic success might reflect the reality that Chinese parents and teachers often ignore the social integration of their young teenagers through sports (Stefansen et al., 2018).

Second, the findings for a common developmental trend in both perceived school influence and community influence, and a negative covariance between the initial status and the rate of change conflicts with the larger body of evidence (Leo et al., 2022). However, the finding that perceived influence decreases over time in or out of school has also been reported in other studies (Dorsch et al., 2022). Given the Chinese physical education backgrounds, most teachers tend to exert psychological pressure toward their students to enforce participation (Aartun et al., 2022); such need-thwarting teachers are more likely to recruit disciplinary measures and criticism toward students who fail to meet their expectations. Those autonomy-thwarting styles may be accounted for slowing the increase of their perceived influence. Thus, to understand the decreased perceived influence in their schools, one must take into account reciprocal processes between youths’ own participation in sports and the general faculty role not limited to physical education teachers. However, in line with the studies of school habitus in China which emphasized academic success so strongly, we thus suggest that the family sport intervention, especially the early socialization process within the family, is the main initiator of this unbalanced reality.

Third, the analyses also provided support for the importance of contextual factors such as personal health condition and time spent on physical activities in predicting adolescent PH. The family social economic status, personal health condition, and weekly time spent on physical activities all positively impacted the initial status of PH. Changes in family social economic status affected the development of PH positively, whereas the effect of changes in health condition and weekly time spent on physical activities were not significant in predicting PH. At the same time, however, the perceived influence of gender in predicting PH becomes more important, as was shown by the current results. The initial level of PH for male students was higher at 38.70% than female students, while there was no significant mean difference in the rate of growth for both. It may be explained by participation in sport, which is constructed socially reported in other researches. Participation in sport is unequivocally stereotyped with gendered habitus favoring specific groups. Sport encourages masculinity and typical masculine characteristics such as speed, strength, and competitiveness (Anderson and White, 2010) and discourages particular forms of femininity (Preece and Bullingham, 2022). These dispositions are often reinforced within repeated practice, solidifying masculine dominance and gendered habitus within family, school, and community. Therefore, it is difficult for teachers or parents to assist students in obtaining required various types of capital which challenge gendered stereotypes and encourage equality.

## Conclusion

Our main contribution is to show initiation in the relevance of family, school, and community sport influence for sport participation across elementary, middle, and high school through a solid retrospective survey material including the whole population of youth—not only elite aspiring young athletes that has been the target group in many previous studies. By this, we add to the growing field of studies on family−school−community relationships in sport. The observed interactional effects that sport influence in their family, school, and community on physical habitus across study stages are explained by Bourdieu’s habitus theory. We further suggest that even though there are interactional effects among family, school, and community with reference to early youth sport socialization, the way actually operates in families might be more important than both left and push gradual changes of school and community toward youth sport socialization. Our contribution is to bring these questions to the forefront and to deliver background knowledge that shows the importance of the interactional effect of sport influence from family, school, and community through the teenage years. Those three parts are frequently thought to function less closely in China.

### Limitations and future research

The current study could be strengthened in the following manners. First, the retrospective investigation was conducted instead of the longitudinal survey, which perhaps over-estimated the correlations among perceived influences. Thus, the findings, especially various perceived influences’ variance, should be interpreted with caution. Second, though perceived influence was measured by self-perception in the present study, which is more reliable than sport performance measures, to some extent, self-assessment by subjects may not properly detect the physical capital influence around them. Therefore, more types of data, such as behavioral data, should be considered in future studies when examining physical capital influence. Third, multivariate LGCM models were built upon the assumption of linearity. Therefore, our models were not able to reflect more complex relationships such as quadratic and cubic relationships. However, prior to modeling and during the preliminary data analyses on the quality of data, we tested the linearity assumption by examining the histograms and scatterplots, and no non-linear relationships were detected. Clearly, the effect of physical capital influence on students’ PH in general, and participatory behavior in particular, is an important topic, and there is still much to be learned in this area.

## Data availability statement

The original contributions presented in the study are included in the article/supplementary material, further inquiries can be directed to the corresponding authors.

## Ethics statement

The studies involving humans were approved by the Chongqing University Institutional Review Board (IRB). The studies were conducted in accordance with the local legislation and institutional requirements. The participants provided their written informed consent to participate in this study.

## Author contributions

JB: Conceptualization, Data curation, Formal analysis, Investigation, Methodology, Project administration, Software, Supervision, Validation, Visualization, Writing – original draft, Writing – review & editing. ZX: Conceptualization, Data curation, Formal analysis, Funding acquisition, Investigation, Methodology, Project administration, Resources, Software, Supervision, Validation, Visualization, Writing – original draft. XX: Data curation, Investigation, Methodology, Software, Supervision, Writing – original draft, Writing – review & editing.
